# ATP Dependent Rotational Motion of Group II Chaperonin Observed by X-ray Single Molecule Tracking

**DOI:** 10.1371/journal.pone.0064176

**Published:** 2013-05-29

**Authors:** Hiroshi Sekiguchi, Ayumi Nakagawa, Kazuki Moriya, Koki Makabe, Kouhei Ichiyanagi, Shunsuke Nozawa, Tokushi Sato, Shin-ichi Adachi, Kunihiro Kuwajima, Masafumi Yohda, Yuji C. Sasaki

**Affiliations:** 1 CREST Sasaki Team, Japan Science and Technology Agency, The University of Tokyo, Kashiwa city, Chiba, Japan; 2 Japan Synchrotron Radiation Research Institute, Sayo, Hyogo, Japan; 3 Foundation Advanced Technology Institute, Tokyo, Japan; 4 Department of Biotechnology and Life Science, Tokyo University of Agriculture and Technology, Tokyo, Japan; 5 Okazaki Institute for Integrative Bioscience and Institute for Molecular Science, National Institute of Natural Sciences, Okazaki, Japan; 6 Department of Functional Molecular Science, School of Physical Sciences, Graduate University for Advanced Studies (Sokendai), Okazaki, Japan; 7 Graduate School of Frontier Sciences, The University of Tokyo, Kashiwa city, Chiba, Japan; 8 High Energy Accelerator Research Organization, Tsukuba, Ibaraki, Japan; 9 PREST, Japan Science and Technology Agency, Kawaguchi, Saitama, Japan; Consejo Superior de Investigaciones Cientificas, Spain

## Abstract

Group II chaperonins play important roles in protein homeostasis in the eukaryotic cytosol and in Archaea. These proteins assist in the folding of nascent polypeptides and also refold unfolded proteins in an ATP-dependent manner. Chaperonin-mediated protein folding is dependent on the closure and opening of a built-in lid, which is controlled by the ATP hydrolysis cycle. Recent structural studies suggest that the ring structure of the chaperonin twists to seal off the central cavity. In this study, we demonstrate ATP-dependent dynamics of a group II chaperonin at the single-molecule level with highly accurate rotational axes views by diffracted X-ray tracking (DXT). A UV light-triggered DXT study with caged-ATP and stopped-flow fluorometry revealed that the lid partially closed within 1 s of ATP binding, the closed ring subsequently twisted counterclockwise within 2–6 s, as viewed from the top to bottom of the chaperonin, and the twisted ring reverted to the original open-state with a clockwise motion. Our analyses clearly demonstrate that the biphasic lid-closure process occurs with unsynchronized closure and a synchronized counterclockwise twisting motion.

## Introduction

Chaperonins are essential ATP-dependent protein-folding machines with a characteristic double-ring structure; each ring is composed of 7–9 subunits of approximately 60 kDa [1], [2]. Each subunit consists of three domains: an apical domain for substrate protein binding, an equatorial domain containing an ATP-binding and inter-ring contact site, and an intermediate domain connecting the apical and equatorial domains by flexible hinges. Chaperonins consist of two sub-families (groups I and II) based on their structure and amino acid sequences [3], [4]. Group I chaperonins, represented by GroEL of *Escherichia coli*, are found in bacteria, mitochondria, and chloroplasts and facilitate protein folding with a co-chaperonin, Hsp10 or GroES, which forms the lid of the folding cage. Group II chaperonins are found in Archaea or in the eukaryotic cytosol, and the built-in lid is formed by helical protrusions in the apical domain. The crystal structures of the closed conformation and biochemical studies suggest the following basic mechanism: the group II chaperonin captures an unfolded protein in the central cavity in an open state, and folding is mediated by ATP-dependent closure of the lid. The folded protein is released from the cavity by opening of the lid. ATP-induced structural changes are essential for chaperonin foldase activity; the closure and opening of the chamber are therefore the focus of group II chaperonin studies [5]–[12].

Recent cryo-electron microscopy (cryo-EM) studies with single-particle reconstruction demonstrate that group II chaperonins twist to seal the central cavity [13], [14]. Zhang et al. determined the structure of a group II chaperonin from a mesophilic archaeon, *Methanococcus maripaludis* (Mm-cpn), in the ATP-free open, ATP-bound intermediate, and ATP-hydrolysis closed states by cryo-EM single-particle analysis [15]. ATP binding causes conformational changes in each chaperonin subunit, leading to slight closure with a 45° (0.785 rad) counterclockwise (CCW) rotation of the apical domain. ATP hydrolysis subsequently drives each subunit to move toward the folding chamber, closing the lid completely. Although there is evidence supporting the conformational changes of group II chaperonins, information concerning their dynamics, such as speed, direction, and timing of the ring’s twisting, is limited. Such dynamics are probably too small to detect with visible-light technology.

In this study, we demonstrate ATP-dependent dynamic motion of a group II chaperonin from *Thermococcus* strain KS-1 (TKS1-Cpn) at the single-molecule level with highly accurate rotational axes views with a refined X-ray technology, diffracted X-ray tracking (DXT). DXT is a powerful technique for detecting subtle dynamic motions of functional proteins at the single-molecule level [16]–[18]. The dynamics of a single protein can be monitored by the trajectory of the Laue spot of a nanocrystal that was labeled on the target protein immobilized on the substrate surface [16]. A remarkable feature of DXT is its ability to monitor the rotational motion of the nanocrystal at several mrad angle scales with two rotational axes views, twisting and tilting motions. Group II chaperonins are expected to undergo rotational motion during the ATP cycle; therefore, the DXT technique could be a suitable tool to monitor ATP-induced dynamics of the chaperonin at the single-molecule level. In this study, UV-light-triggered DXT with caged-ATP and stopped-flow fluorometry were utilized to track the ATP-induced dynamics of a group II chaperonin. The chaperonin’s lid closed partially within one second after ATP binding, the closed ring twisted counter clock-wisely from the top to bottom view of the chaperonin, and the twisted ring turned back to the original open state with a clockwise twisting motion. Our analyses with precise rotational and macroscopic views of chaperonin dynamics demonstrate that there are distinct two modes in the lid-closure process: unsynchronized closure and a synchronized counterclockwise twisting motion.

## Results

### DXT Analysis of the ATP-induced Twisting Motion of Chaperonin

**Figure 1:**
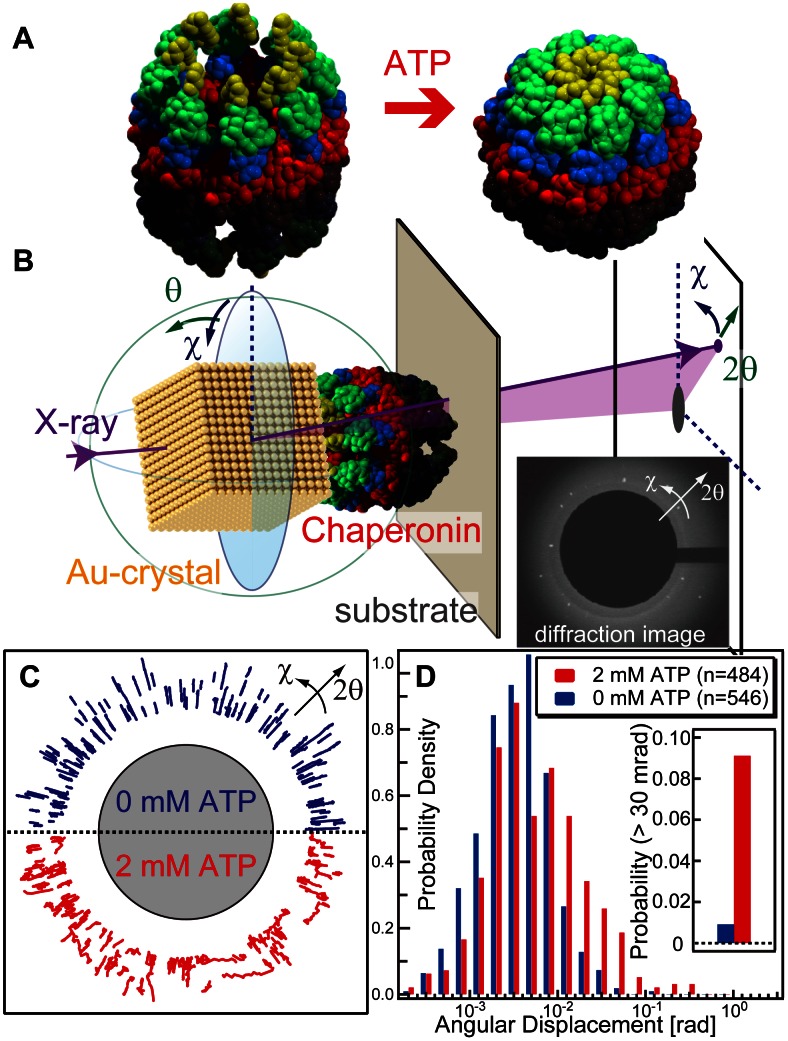
ATP-dependent rotational motion of a group II chaperonin tracked by DXT. (A) Conformational changes of the group II chaperonin in the absence (left) and presence (right) of ATP. (B) Schematic illustration of the detection of internal motions of group II chaperonins by DXT. (C) Typical DXT traces of gold nanocrystals immobilized on the ring of the group II chaperonin in the absence (upper panel) and presence of ATP (lower panel). (D) The distribution of the absolute angular displacement of the group II chaperonin in the twisting (χ) direction. About 500 DXT trajectories are used to make histogram. The trajectories with an angular displacement greater than 30 mrad in the χ direction were counted as inset bar-graph.

Figure 1-A illustrates the model for the conformational change of TKS1-Cpn, which was constructed from the TKS1-Cpn crystal structure in closed conformation [19] and the open structure of Mm-cpn. We used the group II chaperonin TKS1-Cpn because it exhibits high protein folding activity and structural stability [20]. DXT was used to detect intra-dynamic motions at the single-molecule level for the group II chaperonin (Figure 1-B). Gold nanocrystals were used as a tracer of the chaperonin’s motion. The twisting and tilting of the chaperonin correspond to Laue spots from the gold nanocrystal in the concentric circle (χ) and radial (θ) directions, respectively. A mutant, TKS1-Cpn D263C/C366S, which contains only one Cys residue at the tip of the helical protrusion, was immobilized on a gold-coated substrate surface and labeled with a gold nanocrystal through the formation of a gold-thiol bond. The height distribution observed in the AFM image demonstrated that the chaperonin molecules are immobilized on the gold substrate surface in a correctly assembled manner (Figure S1). Since the cysteine residues are introduced on the tip of helical protrusions of chaperonin ring, we think that most of chaperonins are immobilized on gold surface in upright manner. The size distribution of the gold nanocrystals ranged from 20 to 70 nm (Figure S2), comparable to the size of the chaperonin. Therefore, it is plausible that most of the gold nanocrystals interact with multiple cysteine residues in a ring, as shown in Figure 1-B. Therefore, the motion of the gold nanocrystal should reflect the movement of the chaperonin ring. The distances between anchoring points are fixed, and any events that would stretch or reduce the distances between anchoring points are invisible in our DXT experiments. Therefore, original dynamic motion of chaperonin ring might be affected by the gold nanocrystal in some extent, the motion of the chaperonin ring is observed maintaining the initial anchoring distances between Cys residue introduced on lid of chaperonin and gold nanocrystal. Figure 1-C shows typical traces of Laue spots from gold nanocrystals on the chaperonin in the presence of 0 mM (blue lines, upper panel) and 2 mM ATP (red lines, lower panel). Typical time-resolved diffraction images obtained in the absence or presence of ATP are shown in Video S1. In the absence of ATP, most spots moved radially (θ), while movement was both concentric (χ) and radial in the presence of ATP. Figure 1-D shows the distribution of absolute angular displacement in twisting (χ) direction. An angular displacement larger than 30 mrad in the χ direction is rarely observed in the absence of ATP. The probabilities of angular displacement larger than 30 mrad in the χ direction were less than 1% in the absence of ATP and approximately 5% in the presence of 2 mM ATP (inset of Figure 1-D). Angular displacement in the χ direction was clearly activated by the addition of ATP.

To examine whether the observed motion is ATP hydrolysis dependent, mean square angular displacement (MSD) in the θ and χ directions were compared between in the presence and absence of potassium ion (Figure S3). As other chaperonins, the ATPase activity of TKS1-Cpn is negligible under the potassium-free conditions. Although θ directional tilting motion was retained irrespective of the presence of ATP and potassium ion, the χ directional twisting motion was observed only in the presence of ATP and potassium ion. Therefore, it is clearly shown that the twisting motion is induced by ATP hydrolysis.

### Twisting Direction Analysis by Light-triggered DXT with Caged ATP

**Figure 2:**
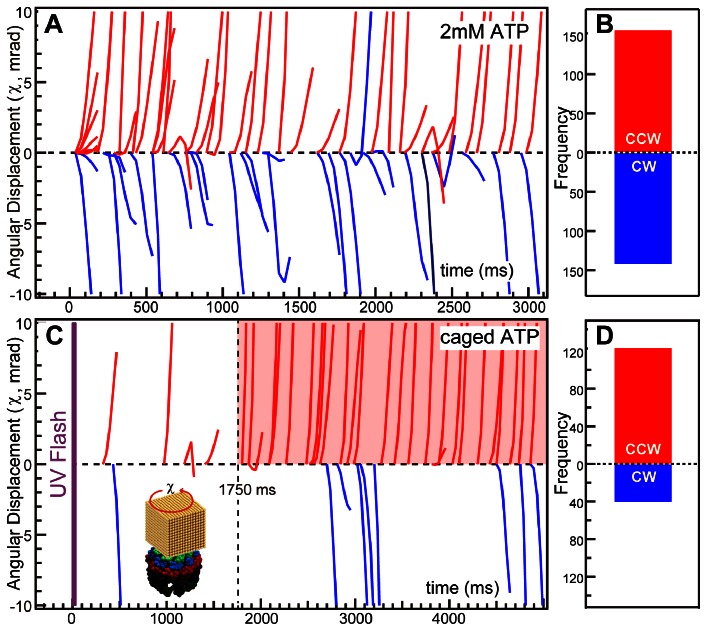
ATP-triggered twisting directional analysis of the group II chaperonin in the χ axis. Rotational position trajectories are shown as a function of time in the presence of 2 mM ATP (A) or 5 mM caged-ATP after a UV flash (C). The histograms in (B) and (D) show the frequency of the initial direction, either clockwise (CW) or counterclockwise (CCW), in the presence of 2 mM ATP or 5 mM caged-ATP, respectively. Trajectories with an angular displacement greater than 30 mrad in the χ direction were selected for inclusion in these figures. The time range with CCW motion of gold nanocrystal in high frequency under UV-triggerd DXT experiment was indicated by transparent red color in Figre C.

Figure 2-A shows the twisting directional (χ) trajectories in the presence of 2 mM ATP. Only angular displacements exceeding 30 mrad in the χ direction were shown. The conformational change model suggests that CCW twisting corresponds to closure of the chamber. However, both CCW and clockwise (CW) twists were observed at similar frequencies. In the experiment, TKS1-Cpn was incubated in the presence of excess ATP; thus, it should be in the dynamic equilibrium between open and closed states. We subsequently attempted to observe rotations from the open to the closed state with caged-ATP. Caged ATP is a derivative of ATP that is inactive and does not bind to the ATP binding site of the chaperonin. UV-light exposure releases the modifying group from the caged ATP to release active ATP in less than 10 ms [21]. TKS1-Cpn was incubated with 5 mM caged ATP, and DXT commenced with UV-irradiation to release ATP. Figure 2-C shows the rotational trajectories after UV exposure. Most of the CCW twisting began after an approximately 1.75-second lag time (the time range with transparent red color in Figure 2-C). However, CW motion occurred at a relatively low frequency (Figure 2-D). Thus, we concluded that the CCW motion reflects rotation of the ring caused by lid closure.

### ATP-induced Tilting Motion Analysis with DXT and Stopped-flow Fluorometry

**Figure 3:**
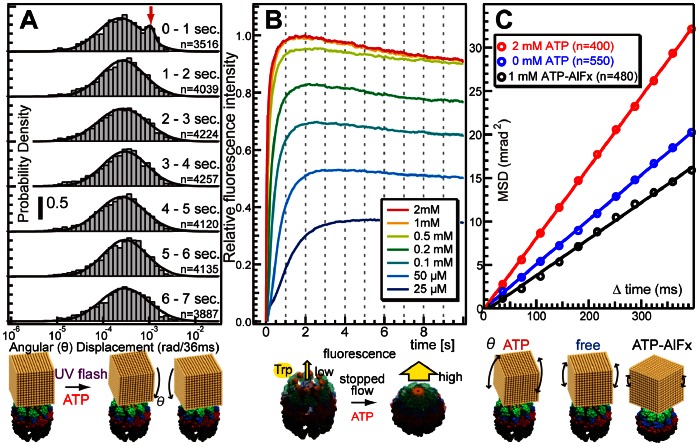
ATP-triggered rotational analysis of group II chaperonins in the θ direction. (A) Time-series histograms of the absolute angular displacement in the θ direction per frame (36 ms). (B) Tryptophan fluorescence changes for a group II chaperonin (TKS1-Cpn L265W) in a mixture of ATP, as measured with a stopped-flow spectrofluorometer. (C) Mean square angular displacement (MSD) in the θ direction as a function of time interval in the presence of 0 mM ATP, 2 mM ATP, or 1 mM ATP-AlFx.

There was a lag between ATP release from the caged ATP and the initiation of CCW motion. Thus, we analyzed the tilting motion (θ) after UV-light exposure. Histograms (Figure 3-A) constructed by recording the segments of displacement each second after UV-light exposure illustrate the absolute angular displacement in the θ direction per frame (36 ms). A bimodal distribution was apparent after 1 s of UV light exposure, while normal distributions were observed at the other times. The fitting parameters of the bimodal or normal distribution in each histogram are shown in Table S1. An additional larger peak at approximately 10^−3^ rad in the first histogram corresponded to a larger tilt of the gold nanocrystal in TKS1-Cpn and could be explained as unsynchronized closure events (inset of Figure 3-A) because the synchronized closure events correspond to translational motions which were not reflected as motion in the θ direction in the diffracted images. The results suggest biphasic conformational changes in group II chaperonins. ATP initially induced movement of each subunit, which was observed as angular displacements in the θ direction, and twisting of the ring is reflected by counterclockwise angular displacement in the χ direction.

Next, we performed stopped-flow fluorometry to macroscopically analyze the kinetics of the ATP-dependent conformational changes by rapid mixing of ATP and the chaperonin mutant TKS1-Cpn L265W, which has only one tryptophan residue at the tip of the helical protrusion. We have previously shown that the addition of ATP enhances the tryptophan fluorescence intensity of TKS1-Cpn L265W, correlating with closure of the lid [5]. The fluorescence change occurred in less than 1 s (Figure 3-B) in the presence of excess ATP, and even when the ATP concentration was low, the change started immediately and nearly completed within 2 s. A similar initial increase in fluorescence at 1 s was observed under potassium-free conditions (Figure S4). As the twisting motion was not observed without potassium ion condition (Figure S3), it is evident that the initial fluorescence change does not correspond to the twisting motion of TKS1-Cpn. Thus, the larger displacement in the θ direction observed by UV light-triggered DXT appears to correspond to the increase of tryptophan fluorescence intensity upon closure. The difference between with and without potassium ion is a gradual decrease of fluorescence after the initial peak in the presence of potassium ion. As the decrease started at the similar timing as the twisting motion, the gradual decrease may correlate with the ATP hydrolysis dependent twisting motion observed by DXT. The abovementioned results clearly correspond to recent observations that ATP binding causes a slight conformational change in each subunit of a chaperonin and subsequent ATP hydrolysis drives each subunit to move toward the folding chamber to close the lid completely [15]. Further motion begins after the initial conformational change of each subunit is completed in 1 s.

Figure 3-C shows the mean square angular displacement (MSD) in the θ direction as a function of time interval in the presence of 0 mM ATP, 2 mM ATP, or 1 mM ATP- aluminum fluoride (AlFx). The linear relationship between MSD and time interval demonstrates that the angular displacement in the θ direction is similar to Brownian motion. The angular diffusion constants estimated from the slope of the MSD curves are shown in Table 1. The slope of the MSD curve obtained in the presence of 1 mM ATP-AlFx is considerably shallower than that obtained in the presence of 0 mM or 2 mM ATP. Incubation with ATP and AlFx traps group II chaperonins in a closed compact conformation [22]. The chaperonin complex in the closed conformation is expected to be more stable than the open conformation, which is confirmed by the normal DXT experiment. The sharp slope in the presence of 2 mM ATP reflects ATP-induced closure events. These results demonstrate that the DXT method is appropriate for monitoring not only the twisting motion (χ-direction) but also the tilting motion (θ-direction) of chaperonins.

**Table 1:** Angular diffusion coefficient of the group II chaperonin in the tilting (θ) direction.

condition	angular diffusion coefficient (rad^2^/sec.)
2 mM ATP	2.04×10^−5^
0 mM ATP	1.28×10^−5^
1 mM ATP-AlFx	1.05×10^−5^

The values were obtained from the slope of the MSD versus time plot (Figure 3-C). The lines in Figure 3-C were fitted with least-squares fitting to the following equation: *MSD = 4Dt*, where *MSD* is the mean square angular displacement, *D* is the angular diffusion constant, and *t* is time interval.

## Discussion

We analyzed ATP-induced structural dynamics of a group II chaperonin (TKS1-Cpn) at the single-molecule level with DXT and at the macroscopic level with stopped-flow fluorometry.

In DXT measurements, the motion of a gold nanocrystal is related to the conformational changes of both rings. It is impossible to dissect these motions in the present experimental setup. The twisting motions of the rings occur in the opposite direction, but their twisting can be monitored in the same directional rotation of the gold nanocrystal because the bottom ring is immobilized on the substrate surface (Figure S5). We cannot exclude the possibility that both rings change their conformations simultaneously. When both rings close or open simultaneously, the gold nanocrystal rotates in the CCW or CW direction, respectively. When the rings are not synchronized, i.e., one ring opens while the other closes, the analysis would be more complicated. Thus it seems not easy to obtain detailed kinetic data. However, in the UV-triggered DXT using caged ATP, only the closing motion should be observed, as the initial conformation is both ring open conformation.

The effect of the gold nanocrystal on the target protein should be considered in the DXT method. Simulation of the molecular dynamics of peptide folding with gold nanocrystals by DXT demonstrated that the nanocrystals affect the protein-folding pathway but not the global-minimum state [23]. In Figure 1-D, only 9% of trajectories exceed 30 mrad angular displacements in χ direction at 2 mM ATP condition. We cannot exclude the effect of the attached gold nanocrystal considering the absolute dynamic parameters, however the extent of the effect was small enough to track the conformational change of chaperonin. Since MSD curves (Figure 3-C) show that the relationships between mean square angular displacement and time interval are liner at all conditions, such as 0 mM ATP, 2 mM ATP and 1 mM ATP-AlFx. If the influence from the attached gold nanocrystal is big enough to alter the dynamics of chaperonin drastically, MSD curve should be saturated at longer lag-time. And, the order of the slope was consistent with the previous result, the slope of the MSD curve obtained in the presence of 1 mM ATP-AlFx was considerably shallower than that obtained in the presence of 0 mM or 2 mM ATP. Furthermore, the twisting motion of gold nanocrystal on chaperonin is observed only in the presence of ATP condition. Therefore, it is reasonable to think that ATP-induced twisting motion of chaperonin was observed at real time. Although the original dynamics of the chaperonin may be affected by the dimension of gold nanocrystal in some extent, the direction of twisting and timing after ATP binding (Figure 2-B) were not affected by the presence of the crystals if you pick up and analyze the traces with larger angular displacement (ex. 30 mrad ) in χ direction. The statistical analysis of DXT was discussed qualitatively (Fig. 3-A and Fig. 3-C). Quantitative analyses will be possible when the technical problems associated with making uniform gold nanocrystals are overcome. The size of the gold nanocrystal distribution is 20 to 70 nm in our current technique (Figure S2).

In addition, there is a risk of X-ray damage to the protein. The photon flux at the sample position was estimated to be approximately 10^15^ photons/sec/mm^2^, and the X-ray spectra are shown in Figure S6. We limited the X-ray exposure time to less than several seconds for each sample according to the previously reported DXT experiments [17], [18].

The ATP hydrolysis cycle of group II chaperonins is notably slow. The wild-type TKS1-Cpn chaperonin hydrolyzes approximately 8 ATP molecules per minute. Only the ATP-dependent motion of the chaperonin was observed in the DXT experiments, and there should be a motionless time during the ATP cycle. The very low frequent CW motions in the experiment with caged ATP support this idea. The observed CW motion may be caused by incomplete ATP reaction cycles or statistically rapid cycles. Although the cause of the CW motion would potentially be clearer if we could use a longer observation time, such a long X-ray exposure would cause significant X-ray damage.

**Figure 4:**
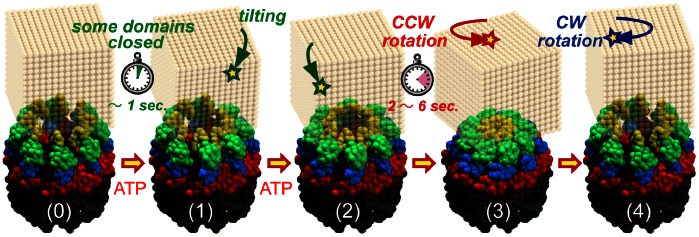
Schematic model of the conformational changes in the group II chaperonin.

Figure 4 is a schematic model of the conformational changes in group II chaperonins based on our results. In the absence of ATP, the chaperonin adopts an open conformation, and the ring fluctuates to some extent (Figure 4-0). The binding of ATP induces a conformational change in each subunit within 1 s (Figure 4-1). Unsynchronized closure events within each subunit occurred during the closing process. Consequently, the ring twisted counterclockwise, viewed from top to bottom, closing completely within 2–6 s (Figure 4-2) and subsequently reverting to the original state by a clockwise twist (Figure 4-3).

In summary, DXT was used to monitor the conformational changes of a group II chaperonin (TKS1-Cpn). Two precise rotational and macroscopic analyses of protein dynamics demonstrated that there are two distinct modes involving closure and twisting motions in the process of lid-closure in group II chaperonins.

The protein folding mechanism of group II chaperonin has been believed to resemble that of group I chaperonin with an only difference in the lid, detachable one and built–in one. However, our results suggest the significant difference between them. Protein folding by group II chaperonin seems to be mediated during the ATP hydrolysis dependent rotational motion, but such rotational motion was not observed in group I chaperonin. The difference might relate with the difference of substrate specificity or function between them. In group II chaperonin, the driving force for protein folding might be given the rotational motion of the cavity. Therefore, the study on the effect of the rotational motion on the captured unfolded protein is indispensable to reveal the protein folding mechanism by group II chaperonin.

DXT was able to track the dynamic motion of the group II chaperonin from *Thermococcus* at the single-molecule level, even at high temperatures, such as 60 °C. DXT eliminated the translational noise that is caused by a temperature change or high temperature in the experimental condition. In addition, high-speed observations, such as sub-μ/frame observations, are also possible with DXT when a high-flux x-ray source and high-speed camera are used. Therefore, our methodology with precise rotational axes views would be a powerful tool to gain insight into the subtle but important dynamic movements of proteins at the molecular level.

## Materials and Methods

### Diffracted X-ray Tracking (DXT)

The dynamics of a single protein were monitored through the trajectories of the Laue spots from the nanocrystal, which was used to label the target protein for DXT [24], [25]. White X-rays, 12–18.5 keV (ΔE/E = 15%, Undulator U20 gap = 15 mm [26]), from the beam line PF-AR NW14 (KEK, Japan) were used to record Laue diffraction spots from the gold nanocrystals on the group II chaperonin. The X-ray beam at the sample was 90 µm (vertical) and 90 µm (horizontal) (full width at half-maximum). The photon flux at the sample position was estimated to be approximately 10^15^ photons/sec/mm^2^, and the X-ray spectra are shown in Figure S6. The diffracted spots from each nanocrystal were monitored with an X-ray image intensifier (V7739P, Hamamatsu Photonics, Japan) and a CCD camera (C4880-80, Hamamatsu photonics). The specimen-to-sample distance was approximately 70 mm and was calibrated by diffraction from gold film. The angular precisions of the DXT in the θ and χ directions were 1 and 5 mrads, respectively. The sample temperature during the DXT was controlled at approximately 60°C by hot air blowers (TRIAC PID, Leister, Switzerland), which is the working temperature of the chaperonin from *Thermococcus* strain KS-1. An Nd-YAG laser (λ = 355 nm, 0.8 mm φ, 10 mJ/cm^2^) was introduced into the X-ray irradiated area for UV-triggered DXT. The sample chamber was replaced after each exposure shot in UV-triggered DXT. Gold nanocrystals were obtained by epitaxial growth on NaCl (100) substrate [27] and were dissolved with detergent (n-decyl-β-D-maltoside (Dojindo Laboratories, Japan), 50 mM MOPS, pH 7.0). The average diameter of the gold nanocrystals was estimated to be 20–70 nm and was confirmed by AFM (Figure S2). Custom software written for IGOR Pro (Wavemetrics, Lake Oswego, OR) was used to analyze the diffracted spot tracks and trajectories.

### Sample Preparation

TKS1-Cpn D263C/C366S in which Asp263 and C366 of wild type TKS1-Cpn were replaced by Cys and Ser, respectively, and TKS1-Cpn L265W in which Leu265 was replaced by Trp were expressed and purified to homogeneity as previously described [5]. A 50-µm-thick polyimide film (Kapton, Du Pont-Toray, Tokyo, Japan) and a 100-µm-thick fluoropolymer film (NEOFLON ETFE film EF-050, Daikin Industries, Osaka, Japan) were coated with chromium (10 nm) and gold (25 nm) by vapor deposition and were used as a substrate surface for DXT and UV-triggered DXT, respectively. An aliquot of a mutant chaperonin solution (0.2 mg/mL) in MOPS buffer (50 mM MOPS, 100 mM KCl, 5 mM MgCl_2_, pH 7.0) was applied to the gold substrate for 8 h at 4°C. The chaperonin-modified surface was rinsed with the same buffer and reacted with gold nanocrystal solution for 8 h at 4°C. The gold nanocrystal-modified chaperonin surface was rinsed with MOPS buffer and stored in the experimental buffer (MOPS buffer with/without ATP) until use. An experimental chamber was constructed of sample substrate film with a spacer of polyimide film of 50-µm thickness. A schematic diagram of the sample holder is shown in Figure S7. The chamber was filled with MOPS buffer containing no ATP, 2 mM ATP, or ATP-AlFx (1 mM ATP, 1 mM Al(NO_3_)_3_, 6 mM NaF) for standard DXT or 5 mM caged ATP (P^3^-[1-(2-nitrophenyl)ethyl]adenosine-5′-triphosphate, Dojindo, Japan) for UV-triggered DXT.

### Stopped-Flow Fluorescence Analysis

The kinetics of ATP-induced structural changes in TKS1-Cpn (L265W) were measured by rapid mixing of equal volumes of ATP and TKS1-Cpn solutions with a stopped-flow spectrofluorometer (SX20, Applied Photophysics). The final concentration of TKS1-Cpn was 0.25 µM. The excitation wavelength was 295 nm, and fluorescence above 320 nm was detected with a 320-nm cut-off filter. A 2-mm path length was used, and the entrance and exit monochrometer slit widths were set to 1 mm. The dead time of the mixing was approximately 2 ms.

## Supporting Information

Figure S1 **AFM analysis of group II chaperonin on gold coated substrate surface.** The sample was prepared as the same manner to the DXT experiment except immobilization of gold nanocrystal. The chaperonin modified surface was imaged in MOPS buffer by tapping mode (MM-AFM NanoScopeIIIa, Veeco co.) and uniformly distributed circular dots were identified as chaperonin (A). The dot’s height was distributed as 13.1+/−2.9 nm (B) and the value was compared favourably with reported height value of 15 nm in closed conformation of KS-1 CPN.
(TIF)

Figure S2 **AFM analysis of gold nanocrystal fabricated on NaCl surface.** The AFM image of gold nanocrystal fabricated on NaCl surface (inset) was obtained by tapping mode in air (MM-AFM NanoScopeIIIa, Veeco co.), and height of the gold nanocrystal was analysed using Gwyddion software (Czech Metrology Institute). The size of gold nanocrystal distributed from 20 to 70 nm.
(TIF)

Figure S3 **Mean square angular displacement (MSD) of chaperonin in the presence and absence of potassium ion.** MSD in the θ (left, tilting) and χ (right, twisting) directions as a function of time interval under 0 mM ATP, 0.1 mM ATP in the presence or absence of potassium ion conditions by DXT method. DXT experiment was performed at BL40XU (SPring-8, Japan) and its details are described in Text S1. The larger twisting motion (χ) was observed in the presence of ATP and potassium ion. Angular diffusion coefficients are obtained from the slope of the MSD versus time plot and those values are shown in Table S2.
(TIF)

Figure S4 **Tryptophan-fluorescence change of group II chaperonin in a mixture of ATP at potassium-free condition using a stopped-flow spectrofluorometer.** The experimental buffer contains 100 mM NaCl instead of 100 mM KCl. The tryptophan fluorescence increase was observed within several seconds after mixture of ATP and chaperonin (TKS1-Cpn L265W), however no gradual decrease was obtained within 20 sec. after the mixture of ATP and chaperonin solution.
(TIF)

Figure S5 **Twisting motion of gold nanocrystal during closure of chaperonin’s ring.** Gold nanocrystal twists CCW from top to bottom view of chaperonin when each of upper (A) or bottom ring (B) closes and both rings close (C). As the twisting motions of the rings occur in the opposite direction, their rotation can be monitored in the same directional rotation of a gold nanocrystal because bottom ring immobilized on substrate surface. Thus, we can observe the twisting motion except only when one ring opens and the other closes simultaneously. In UV-irradiated DXT experiment, most of chaperonin is in open-open conformation at the beginning of the experiment. An UV-irradiation triggered to increase ATP concentration in the experimental chamber and to shift chaperonin ring to closed conformation. Therefore we can confirm the ATP induced twisting direction in our DXT system.
(TIF)

Figure S6 **Photon flux density profile for the DXT experiment.** Photon flux profiles from PF-AR NW14A in KEK and BL40XU in SPring8 were drawn in blue and red lines, respectively. Photon flux at the sample position was estimated around 10^15^ photons/sec/mm^2^ which was comparable to the previous DXT experiment.
(TIF)

Figure S7 **Schematic drawing of DXT sample holder.** A sample holder was made of sample substrate film with a spacer of polyimide film of 50 µm thickness (Kapton, Du Pont-Toray, Tokyo, Japan). The chamber, 11 mm×11 mm×50 µm, was covered with a 50 µm thick polyimide film and a 100 µm thick fluoropolymer film (NEOFLON ETFE film EF-050, Daikin Industries, Osaka, Japan) for standard and UV triggered DXT, respectively. The chamber was sandwiched by stainless steel frames and was screw-clamped.
(TIF)

Table S1 **Fitting parameters for normal or bimodal distribution of angular displacement (θ) of CPN-KS1 under UV-triggered DXT.** Normal distribution is denoted by N(μ, σ^2^), where μ is the mean and σ^2^ is the variance.
(DOC)

Table S2 **Angular diffusion coefficient of the group II chaperonin in the tilting (θ) and twisting (** ***χ*** **) direction by DXT potassium assay.** The values were obtained from the slope of the MSD versus time plot (Figure S3). The line was fitted with least-squares fitting to the following equation: *MSD = 4Dt*, where MSD is the mean square angular displacement, *D* is the angular diffusion constant, and *t* is time interval.
(DOC)

Text S1 **Supplementary method for DXT Potassium assay at SPring-8.**
(DOC)

Video S1 **Time-resolved diffraction images for gold nanocrystals on TKS1-Cpn obtained in absence and presence of ATP conditions.** Original images are modified to clarify Laue spots from gold crystals. Tracked spots are shown in blue and red open circle in absence and presence of ATP condition, respectively.
(MOV)

## References

[1] Hartl FU, Hayer-Hartl M (2002) Molecular chaperones in the cytosol: from nascent chain to folded protein. Science 295: 1852–1858. 11884745 10.1126/science.1068408

[2] Horwich AL, Fenton W a, Chapman E, Farr GW (2007) Two families of chaperonin: physiology and mechanism. Annual review of cell and developmental biology 23: 115–145. 10.1146/annurev.cellbio.23.090506.123555 17489689

[3] Kim S, Willison KR, Horwich AL (1994) Cystosolic chaperonin subunits have a conserved ATPase domain but diverged polypeptide-binding domains. Trends in Biochemical Sciences 19: 543–548. 7846767 10.1016/0968-0004(94)90058-2

[4] Kubota H, Hynes G, Willison K (1995) The chaperonin containing t-complex polypeptide 1 (TCP-1). Multisubunit machinery assisting in protein folding and assembly in the eukaryotic cytosol. European journal of biochemistry/FEBS 230: 3–16. 10.1111/j.1432-1033.1995.tb20527.x 7601114

[5] Iizuka R, Yoshida T, Shomura Y, Miki K, Maruyama T, et al (2003) ATP binding is critical for the conformational change from an open to closed state in archaeal group II chaperonin. The Journal of biological chemistry 278: 44959–44965. 12920124 10.1074/jbc.M305484200

[6] Villebeck L, Persson M, Luan S-L, Hammarström P, Lindgren M, et al (2007) Conformational rearrangements of tail-less complex polypeptide 1 (TCP-1) ring complex (TRiC)-bound actin. Biochemistry 46: 5083–5093. 17417821 10.1021/bi062093o

[7] Stuart SF, Leatherbarrow RJ, Willison KR (2011) A two-step mechanism for the folding of actin by the yeast cytosolic chaperonin. The Journal of biological chemistry 286: 178–184. 21056978 10.1074/jbc.M110.166256 PMC3012972

[8] Meyer AS, Gillespie JR, Walther D, Millet IS, Doniach S, et al (2003) Closing the Folding Chamber of the Eukaryotic Chaperonin Requires the Transition State of ATP Hydrolysis. Cell 113: 369–381. 12732144 10.1016/s0092-8674(03)00307-6

[9] Reissmann S, Parnot C, Booth CR, Chiu W, Frydman J (2007) Essential function of the built-in lid in the allosteric regulation of eukaryotic and archaeal chaperonins. Nature Structural & Molecular Biology 14: 432–440. 10.1038/nsmb1236 PMC3339572 17460696

[10] Bigotti MG, Bellamy SRW, Clarke AR (2006) The asymmetric ATPase cycle of the thermosome: elucidation of the binding, hydrolysis and product-release steps. Journal of molecular biology 362: 835–843. 16942780 10.1016/j.jmb.2006.07.064

[11] Douglas NR, Reissmann S, Zhang J, Chen B, Jakana J, et al (2011) Dual Action of ATP Hydrolysis Couples Lid Closure to Substrate Release into the Group II Chaperonin Chamber. Cell 144: 240–252. 21241893 10.1016/j.cell.2010.12.017 PMC3055171

[12] Iizuka R, Ueno T, Morone N, Funatsu T (2011) Single-Molecule Fluorescence Polarization Study of Conformational Change in Archaeal Group II Chaperonin. PloS one 6: e22253. 21779405 10.1371/journal.pone.0022253 PMC3136518

[13] Booth CR, Meyer AS, Cong Y, Topf M, Sali A, et al (2008) Mechanism of lid closure in the eukaryotic chaperonin TRiC/CCT. Nature structural & molecular biology 15: 746–753. 10.1038/nsmb.1436 PMC2546500 18536725

[14] Zhang J, Baker ML, Schröder GF, Douglas NR, Reissmann S, et al (2010) Mechanism of folding chamber closure in a group II chaperonin. Nature 463: 379–383. 20090755 10.1038/nature08701 PMC2834796

[15] Zhang J, Ma B, Dimaio F, Douglas NR, Joachimiak L a, et al (2011) Cryo-EM Structure of a Group II Chaperonin in the Prehydrolysis ATP-Bound State Leading to Lid Closure. Structure 19: 633–639. 21565698 10.1016/j.str.2011.03.005 PMC3705922

[16] Okumura Y, Oka T, Kataoka M, Taniguchi Y, Sasaki YC (2004) Picometer-scale dynamical observations of individual membrane proteins: The case of bacteriorhodopsin. Physical Review E 70: 021917. 10.1103/PhysRevE.70.021917 15447525

[17] Sagawa T, Azuma T, Sasaki YC (2007) Dynamical regulations of protein-ligand bindings at single molecular level. Biochemical and biophysical research communications 355: 770–775. 17320819 10.1016/j.bbrc.2007.02.031

[18] Shimizu H, Iwamoto M, Konno T, Nihei A, Sasaki YC, et al (2008) Global twisting motion of single molecular KcsA potassium channel upon gating. Cell 132: 67–78. 18191221 10.1016/j.cell.2007.11.040

[19] Shomura Y, Yoshida T, Iizuka R, Maruyama T, Yohda M, et al (2004) Crystal Structures of the Group II Chaperonin from Thermococcus strain KS-1: Steric Hindrance by the Substituted Amino Acid, and Inter-subunit Rearrangement between Two Crystal Forms. Journal of Molecular Biology 335: 1265–1278. 14729342 10.1016/j.jmb.2003.11.028

[20] Kanzaki T, Iizuka R, Takahashi K, Maki K, Masuda R, et al (2008) Sequential action of ATP-dependent subunit conformational change and interaction between helical protrusions in the closure of the built-in lid of group II chaperonins. The Journal of biological chemistry 283: 34773–34784. 18854314 10.1074/jbc.M805303200 PMC3259901

[21] Zscherp C, Barth A (2001) Reaction-Induced Infrared Difference Spectroscopy for the Study of Protein Reaction Mechanisms †. Biochemistry 40: 1875–1883. 11329252 10.1021/bi002567y

[22] Ditzel L, Löwe J, Stock D, Stetter KO, Huber H, et al (1998) Crystal structure of the thermosome, the archaeal chaperonin and homolog of CCT. Cell 93: 125–138. 9546398 10.1016/s0092-8674(00)81152-6

[23] Kawashima Y, Sasaki YC, Sugita Y, Yoda T, Okamoto Y (2007) Replica-exchange molecular dynamics simulation of diffracted X-ray tracking. Molecular Simulation 33: 97–102.

[24] Sasaki YC, Okumura Y, Adachi S, Suda H, Taniguchi Y, et al (2001) Picometer-Scale Dynamical X-Ray Imaging of Single DNA Molecules. Physical Review Letters 87: 248102. 11736543 10.1103/PhysRevLett.87.248102

[25] Sasaki YC, Suzuki Y, Yagi N, Adachi S, Ishibashi M, et al (2000) Tracking of individual nanocrystals using diffracted x rays. Physical review E, Statistical physics, plasmas, fluids, and related interdisciplinary topics 62: 3843–3847. 10.1103/physreve.62.3843 11088902

[26] Nozawa S, Adachi S, Takahashi J, Tazaki R, Guérin L, et al (2007) Developing 100 ps-resolved X-ray structural analysis capabilities on beamline NW14A at the Photon Factory Advanced Ring. Journal of synchrotron radiation 14: 313–319. 17587655 10.1107/S0909049507025496

[27] Okumura Y, Miyazaki T, Taniguchi Y, Sasaki YC (2005) Fabrications of dispersive gold one-dimensional nanocrystals using vacuum evaporation. Thin Solid Films 471: 91–95.

