# Supplementary figures and images for: ATP Dependent Rotational Motion of Group II Chaperonin Observed by X-ray Single Molecule Tracking
Source: PLoS One. 2013 May 29;8(5):e64176. doi: 10.1371/journal.pone.0064176 (PMC3666759; doi:10.1371/journal.pone.0064176)

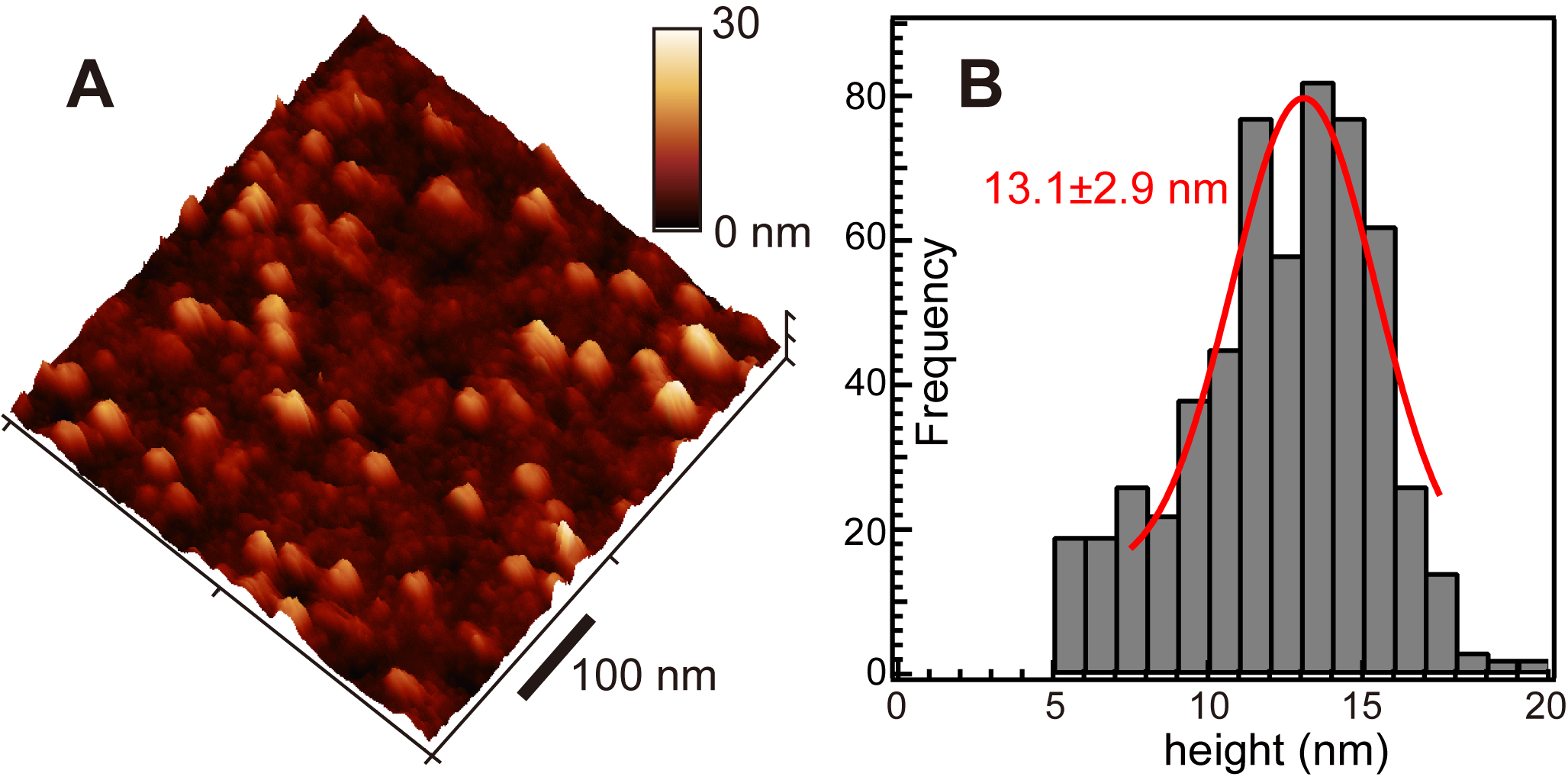

Supplement: Figure S1 — AFM analysis of group II chaperonin on gold coated substrate surface. The sample was prepared as the same manner to the DXT experiment except immobilization of gold nanocrystal. The chaperonin modified surface was imaged in MOPS buffer by tapping mode (MM-AFM NanoScopeIIIa, Veeco co.) and uniformly distributed circular dots were identified as chaperonin (A). The dot’s height was distributed as 13.1+/−2.9 nm (B) and the value was compared favourably with reported height value of 15 nm in closed conformation of KS-1 CPN. (TIF) [file pone.0064176.s001.tif]

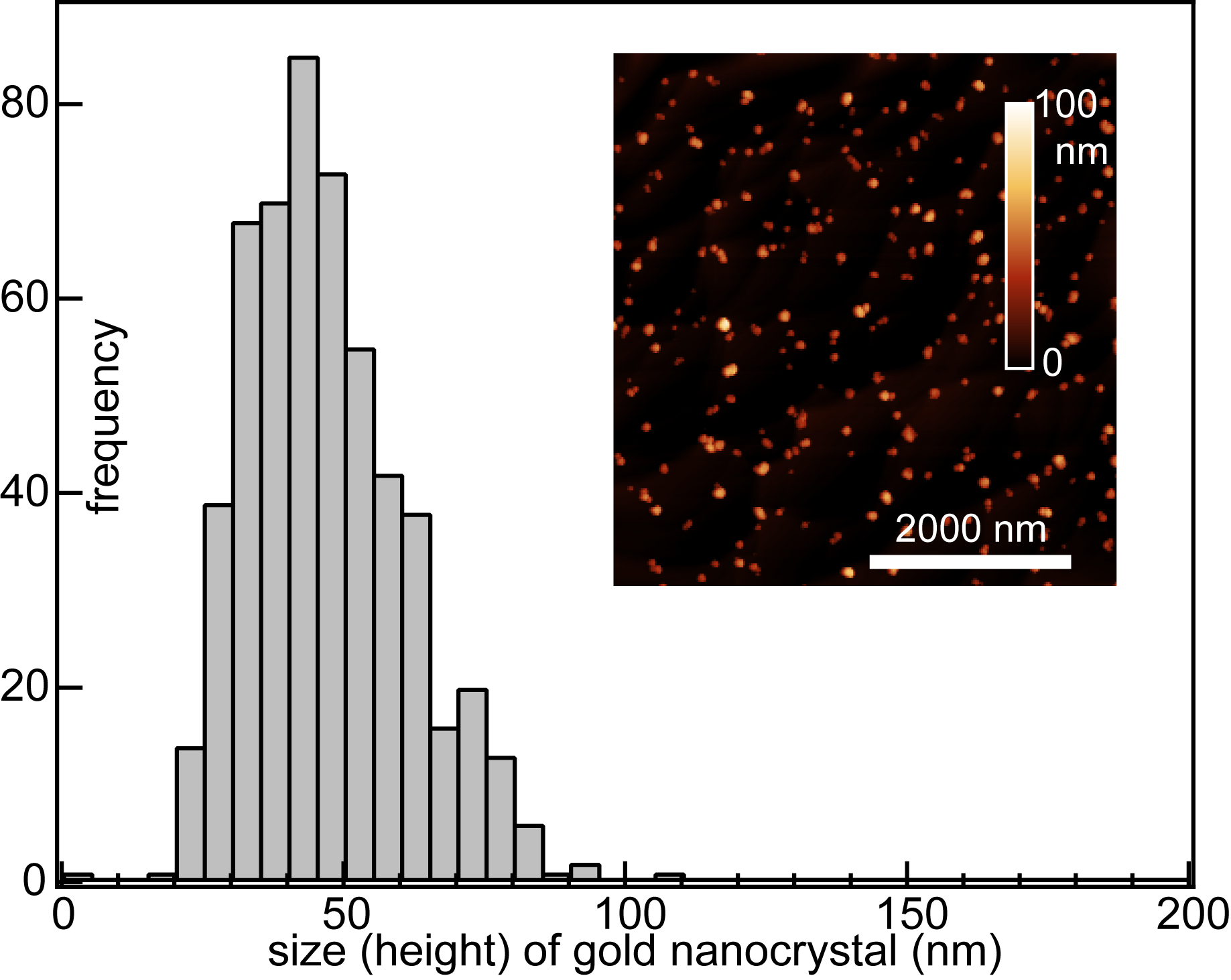

Supplement: Figure S2 — AFM analysis of gold nanocrystal fabricated on NaCl surface. The AFM image of gold nanocrystal fabricated on NaCl surface (inset) was obtained by tapping mode in air (MM-AFM NanoScopeIIIa, Veeco co.), and height of the gold nanocrystal was analysed using Gwyddion software (Czech Metrology Institute). The size of gold nanocrystal distributed from 20 to 70 nm. (TIF) [file pone.0064176.s002.tif]

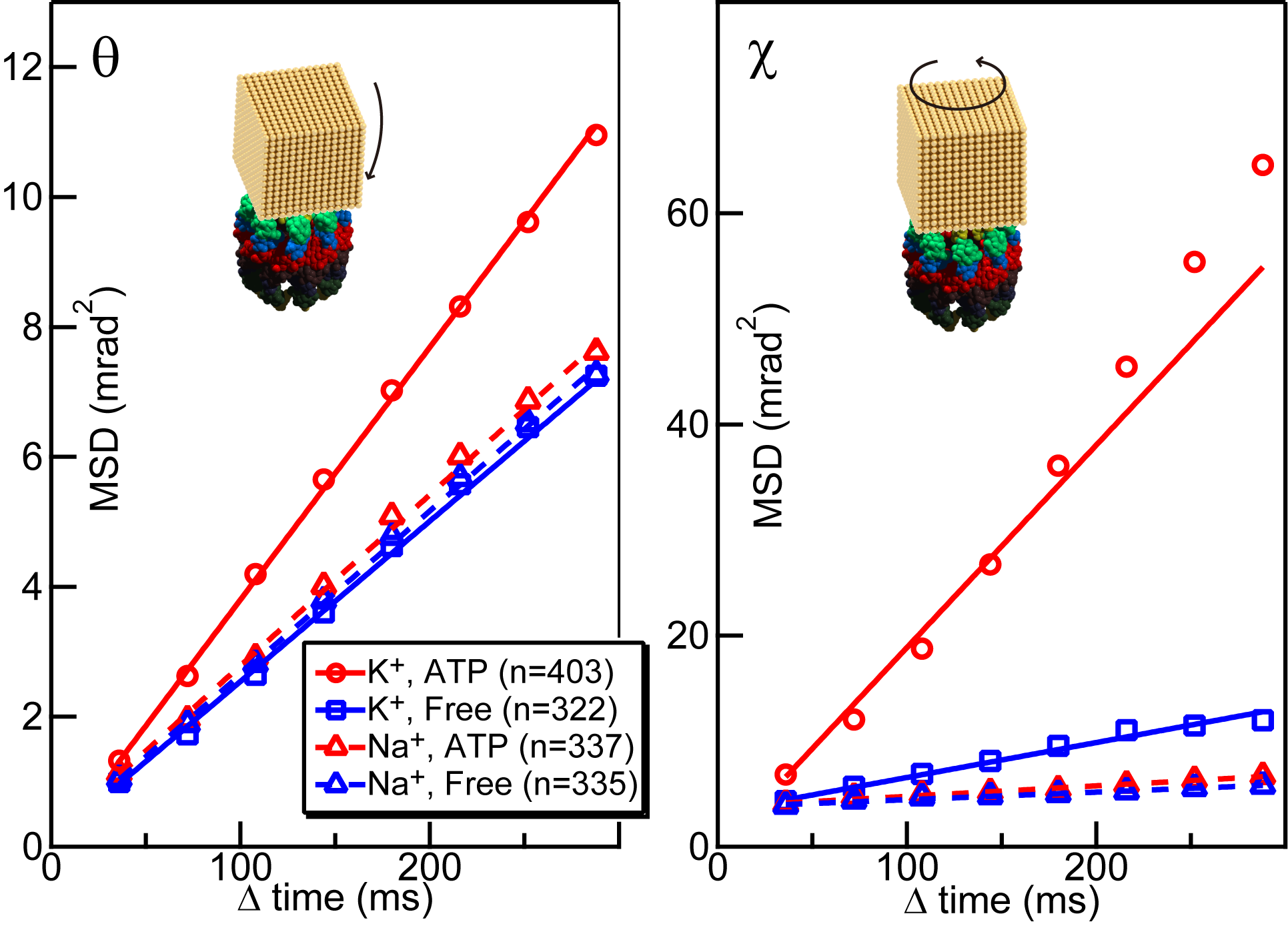

Supplement: Figure S3 — Mean square angular displacement (MSD) of chaperonin in the presence and absence of potassium ion. MSD in the θ (left, tilting) and χ (right, twisting) directions as a function of time interval under 0 mM ATP, 0.1 mM ATP in the presence or absence of potassium ion conditions by DXT method. DXT experiment was performed at BL40XU (SPring-8, Japan) and its details are described in Text S1. The larger twisting motion (χ) was observed in the presence of ATP and potassium ion. Angular diffusion coefficients are obtained from the slope of the MSD versus time plot and those values are shown in Table S2. (TIF) [file pone.0064176.s003.tif]

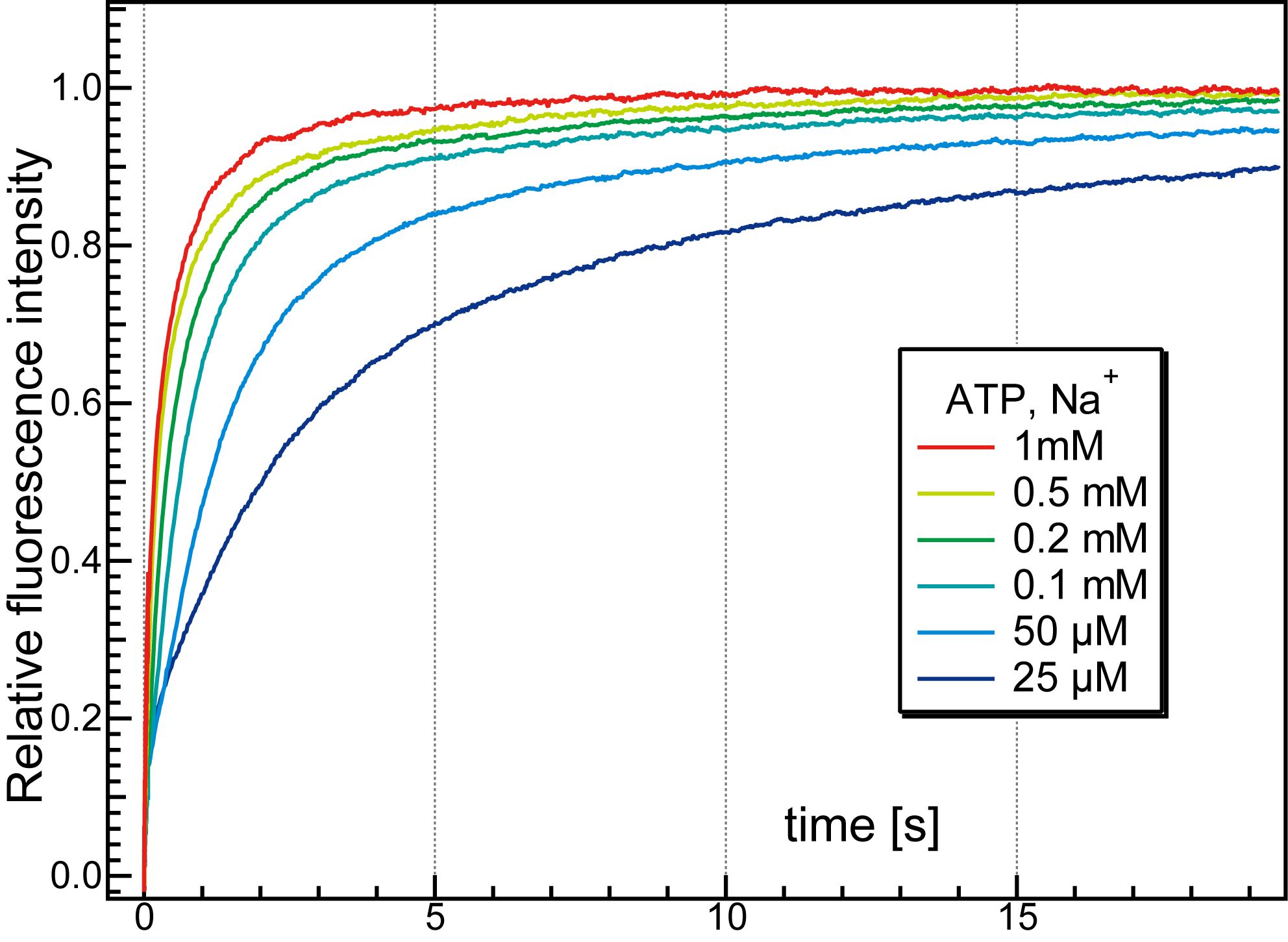

Supplement: Figure S4 — Tryptophan-fluorescence change of group II chaperonin in a mixture of ATP at potassium-free condition using a stopped-flow spectrofluorometer. The experimental buffer contains 100 mM NaCl instead of 100 mM KCl. The tryptophan fluorescence increase was observed within several seconds after mixture of ATP and chaperonin (TKS1-Cpn L265W), however no gradual decrease was obtained within 20 sec. after the mixture of ATP and chaperonin solution. (TIF) [file pone.0064176.s004.tif]

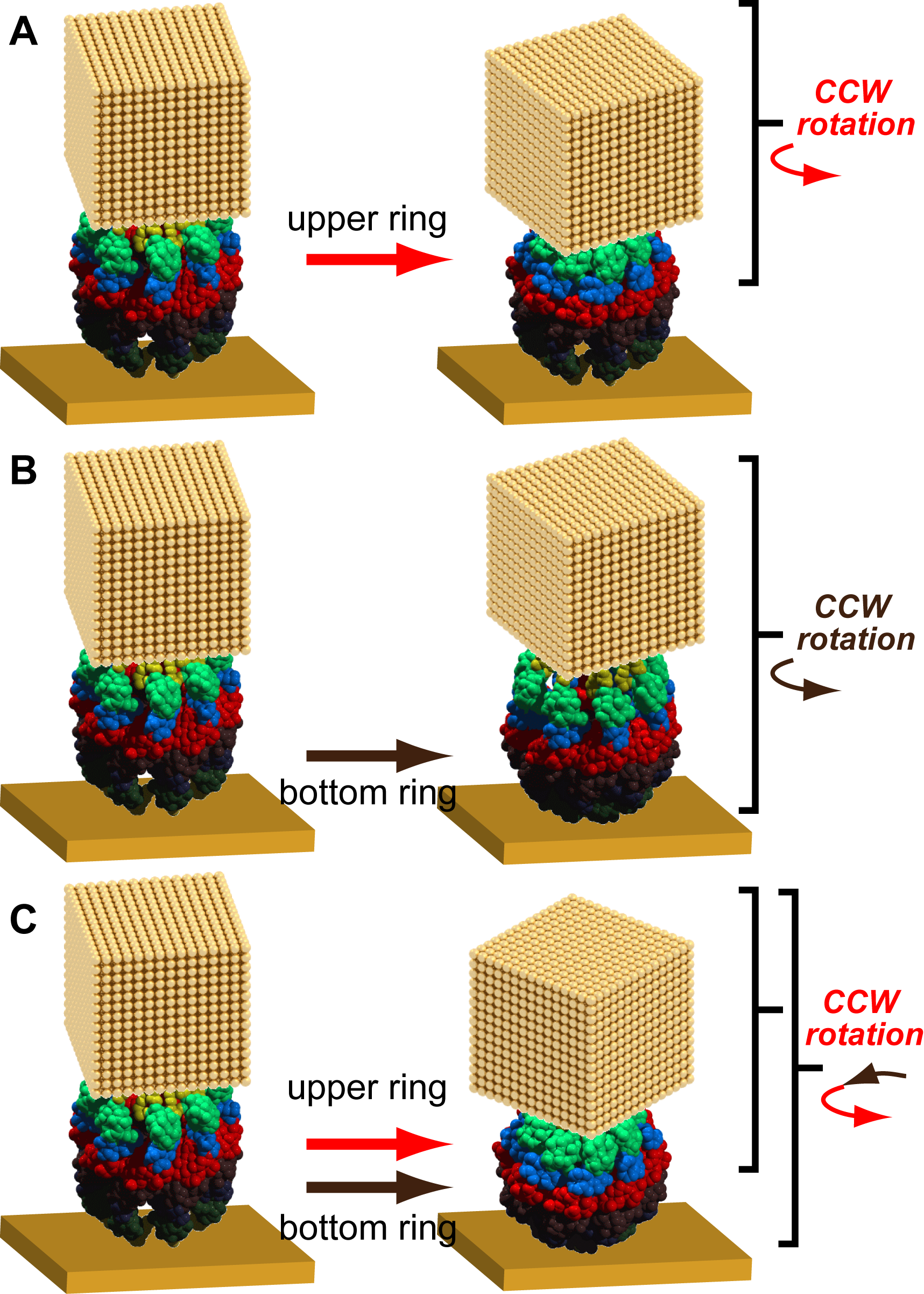

Supplement: Figure S5 — Twisting motion of gold nanocrystal during closure of chaperonin’s ring. Gold nanocrystal twists CCW from top to bottom view of chaperonin when each of upper (A) or bottom ring (B) closes and both rings close (C). As the twisting motions of the rings occur in the opposite direction, their rotation can be monitored in the same directional rotation of a gold nanocrystal because bottom ring immobilized on substrate surface. Thus, we can observe the twisting motion except only when one ring opens and the other closes simultaneously. In UV-irradiated DXT experiment, most of chaperonin is in open-open conformation at the beginning of the experiment. An UV-irradiation triggered to increase ATP concentration in the experimental chamber and to shift chaperonin ring to closed conformation. Therefore we can confirm the ATP induced twisting direction in our DXT system. (TIF) [file pone.0064176.s005.tif]

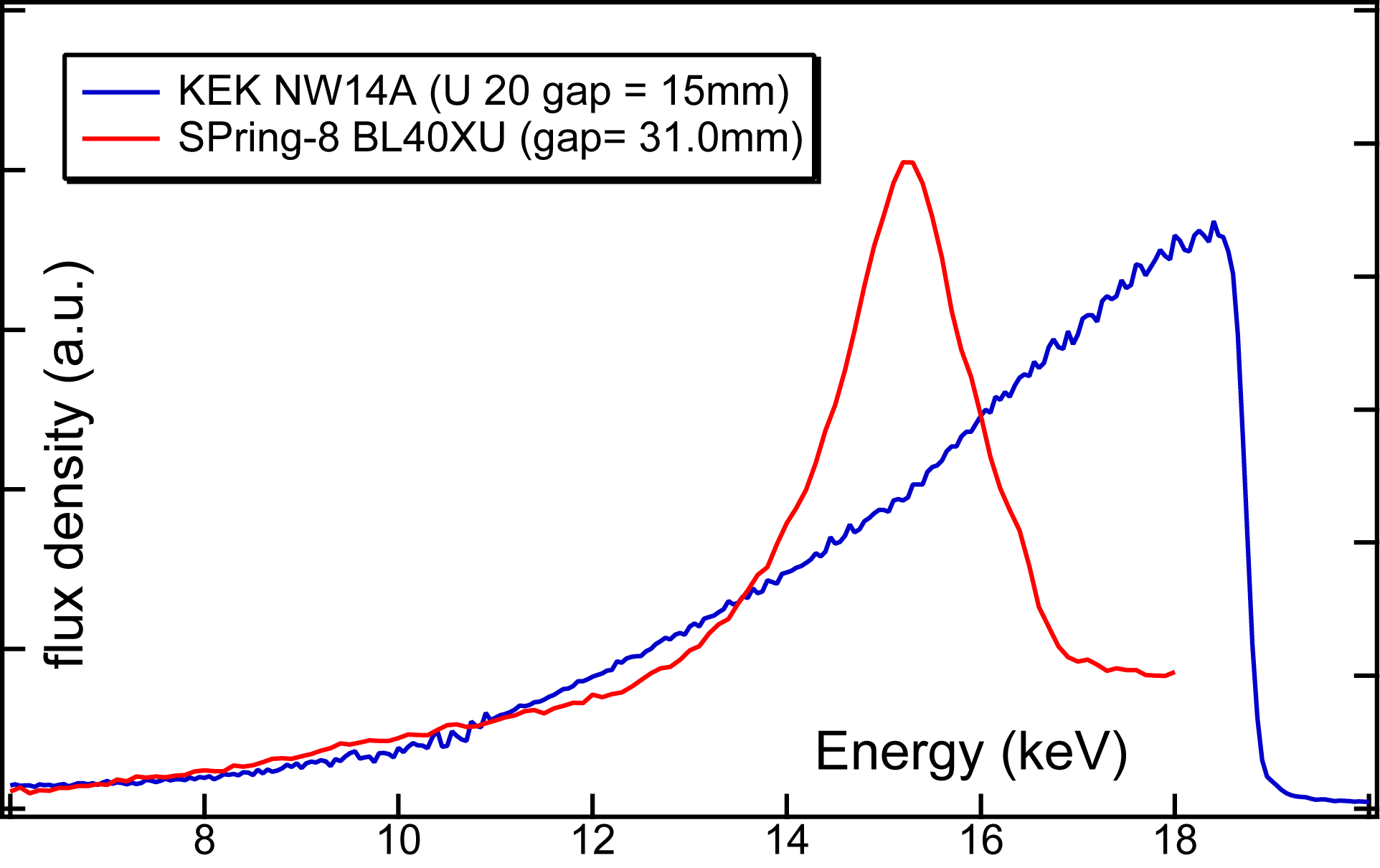

Supplement: Figure S6 — Photon flux density profile for the DXT experiment. Photon flux profiles from PF-AR NW14A in KEK and BL40XU in SPring8 were drawn in blue and red lines, respectively. Photon flux at the sample position was estimated around 1015 photons/sec/mm2 which was comparable to the previous DXT experiment. (TIF) [file pone.0064176.s006.tif]

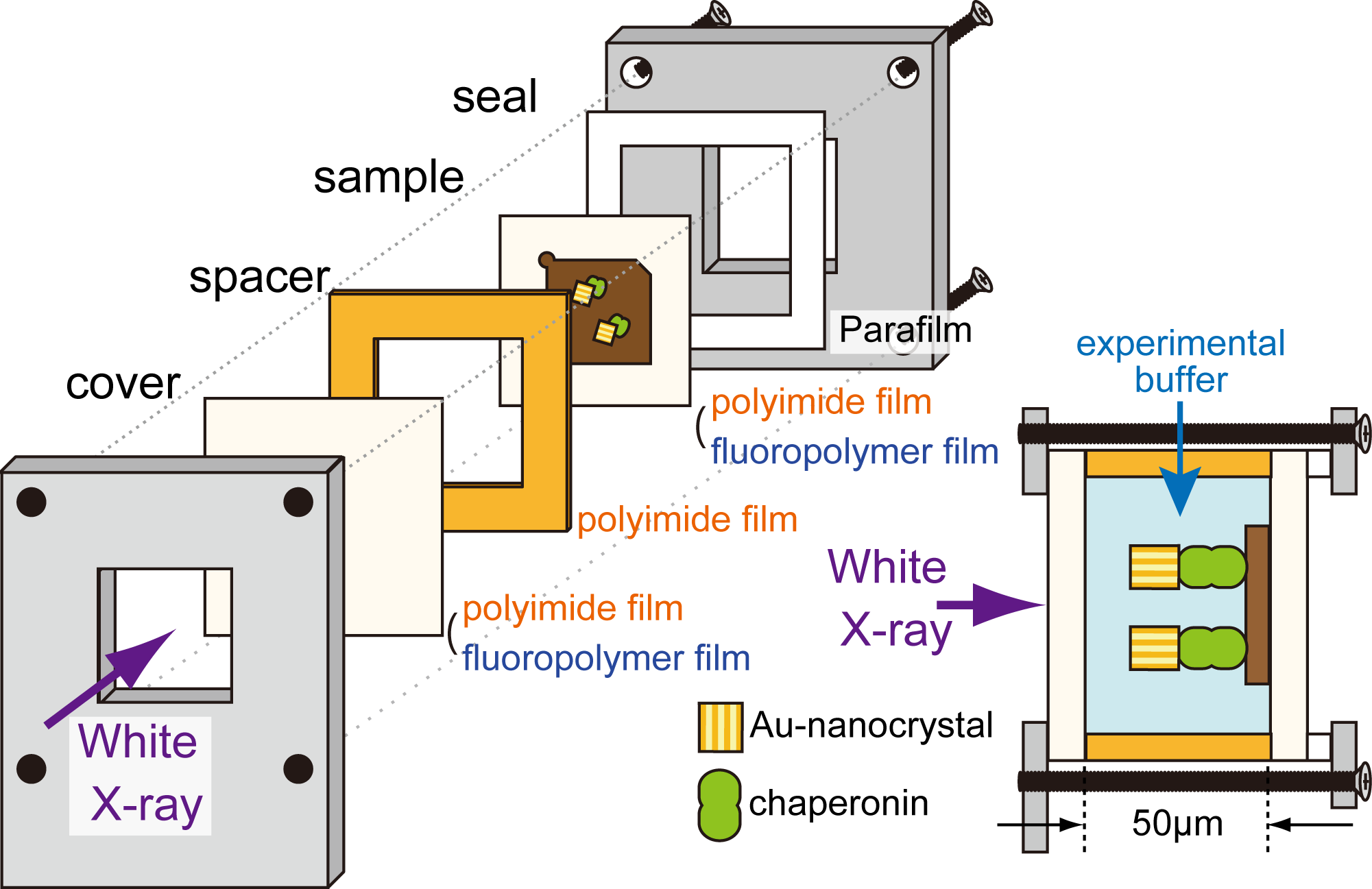

Supplement: Figure S7 — Schematic drawing of DXT sample holder. A sample holder was made of sample substrate film with a spacer of polyimide film of 50 µm thickness (Kapton, Du Pont-Toray, Tokyo, Japan). The chamber, 11 mm×11 mm×50 µm, was covered with a 50 µm thick polyimide film and a 100 µm thick fluoropolymer film (NEOFLON ETFE film EF-050, Daikin Industries, Osaka, Japan) for standard and UV triggered DXT, respectively. The chamber was sandwiched by stainless steel frames and was screw-clamped. (TIF) [file pone.0064176.s007.tif]
