# Supplementary material for: ATP Dependent Rotational Motion of Group II Chaperonin Observed by X-ray Single Molecule Tracking
Source: PLoS One. 2013 May 29;8(5):e64176. doi: 10.1371/journal.pone.0064176 (PMC3666759; doi:10.1371/journal.pone.0064176)
Supplement: Table S1 — Fitting parameters for normal or bimodal distribution of angular displacement (θ) of CPN-KS1 under UV-triggered DXT. Normal distribution is denoted by N(μ, σ2), where μ is the mean and σ2 is the variance. (DOC) [file pone.0064176.s008.doc]

**Table S1: Fitting parameters for normal or bimodal distribution of angular displacement (θ) of CPN-KS1 under UV-triggered DXT.**

| **0 - 1** | *N*(-3.62, 0.49^2^) | N(-2.97, 0.08^2^) |
| --- | --- | --- |
| **1 - 2** | *N*(-3.57, 0.48^2^) |  |
| **2 - 3** | *N*(-3.58, 0.53^2^) |  |
| **3 - 4** | *N*(-3.53, 0.48^2^) |  |
| **4 - 5** | *N*(-3.60, 0.48^2^) |  |
| **5 - 6** | *N*(-3.53, 0.47^2^) |  |
| **6 - 7** | *N*(-3.54, 0.52^2^) |  |

Normal distribution is denoted by N(μ, σ^2^), where μ is the mean and σ^2^ is the variance.
