# Supplementary material for: ATP Dependent Rotational Motion of Group II Chaperonin Observed by X-ray Single Molecule Tracking
Source: PLoS One. 2013 May 29;8(5):e64176. doi: 10.1371/journal.pone.0064176 (PMC3666759; doi:10.1371/journal.pone.0064176)
Supplement: Table S2 — Angular diffusion coefficient of the group II chaperonin in the tilting (θ) and twisting ( χ ) direction by DXT potassium assay. The values were obtained from the slope of the MSD versus time plot (Figure S3). The line was fitted with least-squares fitting to the following equation: MSD = 4Dt, where MSD is the mean square angular displacement, D is the angular diffusion constant, and t is time interval. (DOC) [file pone.0064176.s009.doc]

**Table S2: Angular diffusion coefficient of the group II chaperonin in the tilting (θ) and twisting (*χ*) direction by DXT potassium assay.**

|  | *D_θ_ (rad^2^/sec.)* | *D_χ_ (rad^2^/sec.)* |
| --- | --- | --- |
| K^+^, 0.1 mM ATP | 9.71 × 10^-6^ | 4.79 × 10^-5^ |
| Na^+^, 0.1 mM ATP | 6.57 × 10^-6^ | 2.48 × 10^-6^ |
| K^+^, 0 mM ATP | 6.16 × 10^-6^ | 8.27 × 10^-6^ |
| Na^+^, 0 mM ATP | 6.30 × 10^-6^ | 1.80 × 10^-6^ |

The values were obtained from the slope of the MSD versus time plot (Figure S3). The line was fitted with least-squares fitting to the following equation: *MSD=4Dt*, where MSD is the mean square angular displacement, *D* is the angular diffusion constant, and *t* is time interval.
