# Supplementary material for: ATP Dependent Rotational Motion of Group II Chaperonin Observed by X-ray Single Molecule Tracking
Source: PLoS One. 2013 May 29;8(5):e64176. doi: 10.1371/journal.pone.0064176 (PMC3666759; doi:10.1371/journal.pone.0064176)
Supplement: Text S1 — Supplementary method for DXT Potassium assay at SPring-8. (DOC) [file pone.0064176.s010.doc]

## Supplementary method

**DXT Potassium assay at SPring-8**

DXT experiment was performed in the absence and presence of potassium ion or ATP at BL40XU (SPring-8, Japan). The composition of experimental buffer was based on MOPS buffer (50 mM MOPS, 100 mM KCl, 5 mM MgCl2, pH 7.0) and 100 mM NaCl was dissolved instead of 100 mM KCl. in potassium-free condition. X-rays with broad energy width, 14.0-16.5 keV (Undulator ID gap=31.0 mm, Figure S6), were used to record Laue diffraction spots from the gold nanocrystals on group II chaperonins. The X-ray beam at the sample was 40 μm (vertical) and 250 μm (horizontal). The time-resolved diffraction images were monitored by an X-ray image intensifier (V5445P, Hamamatsu photonics, Japan) and a CCD camera (C4880-80, Hamamatsu photonics). The specimen-to-sample distance was around 100 mm and was calibrated by diffraction from gold film. The sample temperature during DXT was controlled at around 60 °C by hot air blowers (TRIAC PID, Leister, Switzerland).
